# Cross-kingdom quorum sensing: mechanisms driving *Candida albicans* and *Staphylococcus aureus* synergistic pathogenesis

**DOI:** 10.1080/20002297.2026.2744234

**Published:** 2026-11-03

**Authors:** Bowen Guan, Yiyang Li, Qinlu Li, Jingyan Wu, Jialong Yi, Xinyang Li, Jinzhi He, Mingyun Li

**Affiliations:** a State Key Laboratory of Oral Diseases & National Center for Stomatology & National Clinical Research Center for Oral Diseases, Sichuan University, Chengdu, China; b Division of Infection and Immunity, Faculty of Medical Sciences, University College London, London, UK; c Medicine Department, The Royal Marsden NHS Foundation Trust, Chelsea, UK; d Department of Cariology and Endodontics, West China Hospital of Stomatology, Sichuan University, Chengdu, China

**Keywords:** *Candida albicans*, *Staphylococcus aureus*, quorum sensing, co-infection, biofilm, cross-kingdom interactions

## Abstract

**Background:**

Co-infection with *Staphylococcus aureus* and *Candida albicans* represents a prevalent, clinically important polymicrobial challenge that can be associated with worse outcomes than monomicrobial infection. Emerging evidence indicates that their synergistic pathogenicity is shaped by quorum sensing (QS) related and other cross-kingdom interactions.

**Objective:**

To summarize current evidence on cross-kingdom interactions involving the *S. aureus* agr quorum-sensing system and the *C. albicans* farnesol signalling pathway and their contributions to polymicrobial pathogenicity.

**Design:**

This narrative review synthesizes current evidence on QS-related, metabolic, environmental, structural, and host-mediated mechanisms underlying *S. aureus* *-* *C. albicans* interactions.

**Results:**

Extracellular pH regulation, ribose metabolism, mixed-biofilm architecture, and farnesol-induced stress adaptation modulate bacterial virulence and antimicrobial tolerance. Furthermore, microbial signalling and host innate immune responses interact bidirectionally to shape infection outcomes. Importantly, direct recognition of a *C. albicans*-derived quorum-sensing molecule by a defined *S. aureus* QS receptor has not yet been demonstrated.

**Conclusions:**

QS-targeted and anti-adhesion/anti-virulence strategies may offer therapeutic opportunities, but their efficacy requires further validation in host-relevant oral and systemic infection models.

## Key messages

- QS-related, metabolic, environmental, structural, and host-mediated interactions contribute to the synergistic pathogenicity of *Staphylococcus aureus* and *Candida albicans* during polymicrobial infection.
- Metabolic regulation of agr, environmental modification, mixed-biofilm interactions, and farnesol-mediated effects represent mechanistically distinct forms of cross-kingdom interaction and should not all be equated with direct QS signalling.
- Targeting QS-related and dual-species pathogenic interactions may offer therapeutic opportunities, but further validation in host-relevant oral and systemic infections models is required.

## Introduction

Coinfection with *Staphylococcus aureus* and *Candida albicans* represents an important polymicrobial clinical challenge [1–3]. As opportunistic pathogens within the human commensal microbiota, they frequently colonise overlapping host niches (e.g. the oral cavity, skin, and indwelling medical devices) to establish mixed infections, particularly in immunocompromised individuals [2]. Clinical epidemiological data show that 20% of *C. albicans* bloodstream infections are polymicrobial infections, with *S. aureus* ranking as the third most frequently co-isolated bacterial species [3]. This co-existence can alter virulence-related phenotypes of both microorganisms through multiple forms of cross-talk, including enhanced *S. aureus* toxin production and changes in *C. albicans* morphogenesis and cell wall architecture [2]. Concurrently, the resulting polymicrobial biofilms can exhibit greater biomass and enhanced antimicrobial tolerance compared with their single-species counterparts, while co-infection-associated metabolic adaptations can promote microbial survival and immune evasion. These synergistic dynamics can also alter host immune responses and contribute to infection persistence, therapeutic recalcitrance, and increased disease severity and mortality in experimental models [2].

Notably, various experimental models demonstrate that the synergistic pathogenesis of *S. aureus* and *C. albicans* involves the *S. aureus* agr quorum-sensing system and *C. albicans* farnesol-mediated signalling as well as other metabolic, environmental, structural, and host-mediated interactions [4,5]. Despite the important role of QS-related cross-talk in shaping pathogenic outcomes, the precise, dynamic networks governing these cross-kingdom interactions remain fragmented across the literature. This review delineates the interactions involving the *S. aureus* agr system and the *C. albicans* farnesol pathway, while distinguishing QS-related regulation from other forms of cross-kingdom interaction, and highlights their links with biofilm remodelling, metabolic regulation, and oxidative stress adaptation. By integrating these mechanistic insights with host-immune dynamics, we further discuss emerging QS-targeted therapeutic strategies, highlighting potential avenues for addressing the therapeutic challenges associated with these dual-species infections.

## Review methodology and literature selection

This article was conducted as a narrative review. The literature search was performed primarily in PubMed, with Web of Science used as a complementary database. The search was updated on 14 September 2026, with no lower publication-date limit imposed. Principal search terms included ‘Candida albicans,’ ‘Staphylococcus aureus,’ ‘quorum sensing,’ ‘agr,’ ‘accessory gene regulator,’ ‘farnesol,’ ‘cross-kingdom,’ ‘interkingdom,’ ‘biofilm,’ ‘co-infection,’ ‘coinfection,’ ‘polymicrobial,’ ‘metabolism,’ ‘adhesion,’ ‘virulence,’ and ‘host immunity.’ Additional targeted searches were conducted for specific topics addressed in the review, including AgrD/AIP maturation and MroQ, RNAIII-mediated virulence regulation, farnesol signalling, oral co-colonisation and denture-associated biofilms, oral mucosal infection, host–microbe interactions, and QS-related therapeutic strategies. Search terms were adapted as appropriate for each database, and the reference lists of relevant primary studies and reviews were also examined to identify additional publications. Studies were selected on the basis of their relevance to the mechanistic, ecological, clinical, or therapeutic aspects of *C. albicans-S. aureus* interactions or to the individual signalling pathways required to interpret these interactions. Primary studies were preferentially used to support mechanistic claims, whereas review articles were used mainly for background context and identification of additional literature. Evidence from in vitro, ex vivo, animal, and human studies was considered according to its experimental context. Duplicate records, conference abstracts, and publications outside the scope of the review were not considered. Given the mechanistic breadth and heterogeneity of the available evidence, the literature was synthesised narratively rather than as a systematic review or meta-analysis.

## Oral ecological context and clinical relevance

The oral cavity provides multiple ecological opportunities for *C. albicans* and *S. aureus* to coexist. In denture wearers, the two organisms have been detected together on both the oral mucosa and prosthetic surfaces [4], and both are frequently recovered from the oral mucosa of patients with denture stomatitis. Oral MRSA–*Candida* spp. co-colonisation has also been documented in immunocompromised children receiving chemotherapy [6]. *S. aureus* can be carried independently in the oral cavity, supporting the mouth as an additional reservoir alongside the nasal niche [7]. The oral ecosystem is highly heterogeneous, comprising distinct soft-tissue habitats and non-shedding surfaces such as teeth and removable prostheses; the latter provide a favourable niche for denture-associated biofilm accumulation and persistence. Oral microbial communities are further shaped by dynamic nutrient availability, salivary factors, and local pH conditions [8]. Oral mucosal models further show that both organisms can invade oral tissues and induce inflammatory responses, although dual infection does not necessarily amplify these responses over single-species infection [9]. These oral ecological features provide a clinically relevant setting in which the metabolic, environmental, structural, and host-mediated interactions discussed below may occur, while direct cross-kingdom QS in human oral communities remains to be demonstrated.

## Quorum sensing system of *Staphylococcus aureus* and *Candida albicans*

Quorum sensing (QS) is a core cell-to-cell communication mechanism that enables microorganisms to regulate population density-dependent gene expression through the secretion, detection, and response to signalling molecules known as autoinducers (AIs) [10]. Driven by the AI accumulation concentration and a subsequent threshold-dependent response, this process coordinates collective behaviours across microbial communities, including virulence factor secretion, biofilm formation, antibiotic synthesis and environmental adaptation [11]. Since the early discoveries that laid the foundation for quorum-sensing research in *Streptococcus pneumoniae* and marine *Vibrio* species during the 1960s and 1970s [11], elucidating these signalling networks has become increasingly pivotal for deciphering the complexities of inter-species interactions.

The major QS-related regulatory systems of *S. aureus* include the accessory gene regulator (agr) system, LuxS/AI-2 system, and the proposed RAP-TRAP system [12]. The agr system is the primary QS pathway involved in the regulation of virulence gene expression, whereas the LuxS/AI-2 system has been implicated in signalling and virulence regulation and the proposed RAP-TRAP system has been suggested to the modulation of staphylococcal gene expression and virulence-related behaviours [13,14].

Genomically, the *S. aureus* agr system is encoded by a chromosomal locus containing two divergent transcriptional units, RNAII and RNAIII, driven by the P2 and P3 promoters, respectively [15]. The RNAII (agrBDCA) operon encodes 4 core components: AgrB, AgrD, AgrC, and AgrA. AgrD is the precursor peptide of AIP. Its C-terminal tail is cleaved by the membrane-bound protease AgrB, which promotes formation of the thiolactone ring. MroQ mediates N-terminal leader cleavage during AIP maturation. Together, AgrB-dependent and MroQ-dependent processing generates mature AIP, although the precise sequence and coupling of peptide maturation and export remain incompletely defined [16,17]. Mature extracellular AIP is sensed by the membrane-bound histidine kinase AgrC, whereas AgrA functions as the response regulator [18,19]. Upon reaching a threshold concentration, AIP binding triggers AgrC autophosphorylation, followed by phosphotransfer to AgrA [19]. Phosphorylated AgrA directly binds to the P2 and P3 promoter regions to activate RNAII and RNAIII transcription, respectively, while also directly activating the *psmα* and *psmβ* promoters independently of RNAIII [20]. While RNAII participates in the autoinducing positive-feedback loop that amplifies agr signalling, RNAIII serves as the principal post-transcriptional effector of the agr system [15,20]. It regulates virulence gene expression through antisense base-pairing with target mRNAs, thereby controlling translation initiation at ribosome-binding sites [20]. A representative example is the translational activation of *α*-hemolysin (*hla*), in which RNAIII interaction with the 5′ UTR relieves inhibitory secondary structures and promotes ribosome access [21]. RNAIII also represses surface-associated proteins such as protein A [15]. In addition to direct post-transcriptional control, RNAIII indirectly modulates virulence through transcriptional regulators such as Rot, SarT, and SarS [20]. Collectively, this regulatory programme promotes a shift from the expression of surface-associated colonisation factors toward the production of secreted virulence factors, a hallmark of agr-mediated virulence conversion [15,20]. Integration of AgrC-AgrA signalling with RNAIII-mediated regulation enables coordinated control of staphylococcal virulence gene expression.

*C. albicans* is a polymorphic fungus that utilises QS molecules to regulate morphogenesis, biofilm development, and interactions with the host [22]. Among the best-characterised QS molecules in *C. albicans* are farnesol, a sesquiterpene alcohol, and tyrosol, an aromatic alcohol [22]. Farnesol (C_15_H_26_O; molecular weight, 222.36) remains the most extensively studied fungal QS molecule [22,23]. Farnesol modulates multiple aspects of *C. albicans* physiology, including hyphal development and biofilm formation, and can also exert detrimental effects on host cells [22,23]. Farnesol biosynthesis is linked to the isoprenoid/sterol biosynthetic pathway, with farnesyl pyrophosphate (FPP) serving as its immediate precursor [24]. Dpp3, an isoprenoid phosphate phosphatase encoded by *DPP3*, contributes to the conversion of FPP to farnesol [25]. A *DPP3* knockout mutant produced approximately sixfold less farnesol than the parental strain and showed reduced pathogenicity in a murine model of systemic candidiasis; reconstitution of *DPP3* restored both farnesol production and pathogenicity [25]. More recent genetic evidence indicates that farnesol accumulation and secretion are regulated by a broader transcriptional network, while the mechanisms governing its synthesis, secretion, regulation, and turnover remain incompletely understood [26].

## Cross-kingdom regulation of adhesion and biofilm formation in *C. albicans*-*S. aureus* co-culture

The *S. aureus* agr quorum-sensing system is a major regulator of biofilm development and contributes to the transition between biofilm maturation and dispersal [5,27]. During early biofilm development, relatively low agr activity may favour bacterial attachment and biofilm formation, whereas increased agr activation is associated with biofilm remodelling and dispersal [27–29]. As cell density increases, agr activation promotes biofilm remodelling partly through induction of extracellular proteases, including staphopains, which can degrade proteinaceous components of the biofilm matrix [30]. The agr system regulates biofilm remodelling through multiple downstream mechanisms, including RNAIII-dependent regulation and direct AgrA-mediated control of phenol-soluble modulins (PSMs). AgrA directly activates genes encoding PSMα, PSMβ, and *δ*-toxin [31–34]. These downstream factors contribute to matrix remodelling and release of *S. aureus* cells from mature biofilms. PSMs may facilitate biofilms dispersal by disrupting matrix interactions, while spatial variation in PSM expression promotes characteristic biofilm channels and detachment patterns [31,33–35]. However, the relationship between agr activity and biofilm formation is context-dependent, as agr inactivation can enhance biofilm formation under certain conditions, likely by reducing dispersal-associated process [28,36].

In parallel with agr-mediated regulation of the bacterial biofilm state, farnesol modulates the fungal contribution to biofilm architecture by suppressing *C. albicans* filamentation through modulation of multiple morphogenetic regulatory networks, including the Tup1-Nrg1 repressor system and Ras1-cAMP-Efg1 signalling. These effects are accompanied by reduced expression of hypha-associated genes such as *HWP1* and *RBT1*[37–40]. This farnesol-mediated repression of filamentation decreases hypha-associated adhesion capacity and compromises the structural development of *C. albicans*. In polymicrobial settings, these effects may further influence *S. aureus-C. albicans* biofilm architecture and interspecies interactions, although whether such effects represent direct QS-mediated cross-kingdom signalling or indirect ecological consequences remains to be clarified [2,41,42].

Farnesol can influence *S. aureus* growth, biofilm-associated behaviour, and antimicrobial susceptibility in a concentration-dependent manner. At high concentrations (>100 μM), farnesol inhibits *S. aureus* growth, compromises bacterial viability, and enhances susceptibility to several antibiotics [43]. It may also inhibit bacterial lipase activity, potentially affecting nutrient acquisition and colonisation-associated traits [44]. Both direct effects on bacterial physiology and indirect effects mediated through fungal-bacterial interactions may contribute to these inhibitory effects. On the one hand, farnesol suppresses hyphal development in *C. albicans*, which may reduce the fungal scaffold available for *S. aureus* attachment within mixed biofilms. In addition, farnesol directly affects bacterial physiology by interfering with C55 lipid carrier recycling, impairing peptidoglycan precursor transport, and reducing cell-wall synthesis, thereby increasing susceptibility to *β*-lactam antibiotics [45]. In contrast, lower concentrations of farnesol have been reported to induce adaptive responses in *S. aureus*, including increased antimicrobial tolerance through oxidative-stress responses and upregulation of multidrug-efflux pumps [46]. These adaptations may enhance resistance to hydrogen peroxide-mediated killing and influence bacterial persistence within polymicrobial environments [47]. Importantly, other fungal-derived metabolites, such as prostaglandin E2, can also modulate *S. aureus* growth and biofilm formation, highlighting that interspecies interactions cannot be attributed solely to QS-mediated signalling [48].

Quorum sensing systems contribute to the regulation of mixed *S. aureus-C. albicans* biofilm development and antimicrobial responses [2]. Confocal laser scanning microscopy (CLSM) demonstrates that *S. aureus* preferentially associates with *C. albicans* hyphae, forming bacterial microcolonies through interactions with hypha-associated adhesins, such as Als3 [2,41]. As described above, farnesol-mediated inhibition of *C. albicans* filamentation may indirectly influence adhesion and organisation within mixed-species biofilms. In mixed biofilms, farnesol alters biofilm architecture, reduces hypha-associated microbial organisation, and enhances the activity of selected antimicrobial treatments targeting cell-wall synthesis [42]. Farnesol further modulates the microbial interaction interface by reducing hypha-associated adhesion properties and weakening bacterial-fungal spatial organisation [2,42]. Scanning electron microscopy (SEM) analysis revealed disrupted three-dimensional biofilm structure fragmented hyphal networks, and altered bacterial microcolony distribution following farnesol treatment [42]. These findings provide a rationale for exploring QS-targeted anti-biofilm strategies aimed at disrupting microbial interactions and enhancing antimicrobial efficacy.

## Cross-kingdom metabolic dialogue: how *C. albicans* remodels the microenvironment to modulate *S. aureus* quorum sensing and virulence

During co-infection, *C. albicans* can modulate the *S. aureus* agr system by altering their shared metabolic microenvironment, thereby contributing to synergistic pathogenicity [1,49]. One such mechanism involves environmental pH modulation. *C. albicans* utilises amino acid as alternative carbon sources, with Stp2p regulating amino-acid permeases and catabolic enzymes [50,51]. Amino-acid catabolism is associated with ammonia release and extracellular alkalinization, shifting initially acidic environments toward a more neutral pH [50]. This metabolic buffering promotes *C. albicans* hyphal formation and may also relieve acid-induced repression of the *S. aureus* agr system [50,52,53]. Importantly, agr expression is maximal around neutral pH under controlled culture conditions and decreases markedly under alkaline conditions, indicating that pH-dependent regulation is not simply proportional to alkalinization [53]. Moreover, although *C. albicans*-mediated alkalinization can enhance *S. aureus* agr activity in vitro, an alkalinization-deficient *C. albicans* mutant retained the ability to induce lethal synergism in vivo, indicating that pH modulation alone is insufficient to account for the interaction [1].

More recent evidence has identified nutrient depletion as an additional metabolic mechanism linking *C. albicans* metabolism to *S. aureus* agr activation. Zcf13-dependent ribose utilisation by *C. albicans* reduces extracellular ribose, thereby relieving ribose-mediated repression of the agr system and promoting *α*-toxin production [1]. Consistent with enhanced agr activity during polymicrobial growth, co-culture with *C. albicans* increased *S. aureus* RNAII (2.5-fold), *agrA* (7-fold), *hld* (5.1-fold), and *hla* (80-fold) expression [49]. In the corresponding murine intra-abdominal co-infection model, coinfection caused 80-100% mortality, whereas genetic disruption of *hla* abolished early lethal synergism, demonstrating that *α*-toxin is necessary, although not sufficient, for this phenotype [49].

A distinct mode of cross-kingdom regulation involves *C. albicans*-mediated modulation of the *S. aureus* agr system through extracellular ribose availability. In both co-culture and a murine intra-abdominal infection (IAI) model, the *C. albicans* transcription factor Zcf13p plays a key role in this process [1]. Zcf13p regulates genes involved in pentose metabolism, including *RBK1*, encoding ribokinase, and *HGT7*, encoding a putative low-affinity ribose transporter implicated in ribose uptake [1]. Consistent with this metabolic capacity, *C. albicans* SC5314 has been experimentally shown to utilise D-ribose as a carbon source [54]. Exogenous ribose suppresses the staphylococcal agr system, whereas its uptake and metabolism by *C. albicans* deplete extracellular ribose, thereby relieving this repression and enhancing agr activation [1]. Consequently, deletion of *ZCF13* reduces *RBK1* and *HGT7* expression and impairs fungal ribose utilisation, resulting in extracellular ribose accumulation, sustained agr repression, and reduced *α*-toxin production [1]. These defects attenuate synergistic lethality in the murine IAI model [1]. This metabolic effect is unlikely to result from direct competitive interference with a canonical AI-2 receptor mechanism. Although LuxS/AI-2-dependent regulation of capsular polysaccharide production through the KdpDE system has been reported in *S. aureus* [55], independent experiments found no detectable cross-talk between the LuxS/AI-2 and agr systems and no consistent AI-2-dependent effect on hemolysin production [56].

Beyond the mechanisms described above, metabolic and physicochemical changes in the local microenvironment may further modulate the *S. aureus* agr system and biofilm dynamics. *C. albicans* exhibits substantial metabolic flexibility during infection, including activation of alternative carbon-utilisation pathways such as gluconeogenesis and the glyoxylate cycle [51]. In mixed biofilms, glucose is abundant in the *C. albicans* extracellular matrix and has been proposed to enhance *S. aureus* growth and biofilm formation [2]. Conversely, glucose depletion in established *S. aureus* biofilms activates the agr system and induces agr-dependent biofilm dispersal [29,31]. These observations raise the possibility that *C. albicans*-associated changes in local carbon availability may indirectly influence agr-dependent biofilm dynamics, although this mechanism has not yet been directly demonstrated in mixed-species biofilms.

In addition to nutrient-dependent effects, *C. albicans*-derived farnesol can induce adaptive stress responses in *S. aureus*. Farnesol concentrations of approximately 40 μM, comparable to those detected in *C. albicans* biofilm supernatants, increase intracellular ROS, upregulate drug-efflux activity, and enhance *S. aureus* tolerance to vancomycin [46]. Repeated farnesol exposure also reduces staphyloxanthin production and induces a broad oxidative-stress response, paradoxically increasing bacterial tolerance to H₂O₂ and macrophage-mediated killing [47]. Finally, physical transport conditions further shape agr signalling independently of fungal metabolism. Under fluid flow, autoinducing peptide (AIP) is efficiently advected away from cells near the biofilm-fluid interface but can accumulate at the base of sufficiently thick biofilms, producing spatially heterogeneous agr activation [57].

In conclusion, *C. albicans* modulates the *S. aureus* agr-associated virulence network through multiple mechanisms, including metabolic regulation through ribose depletion, environmental remodelling through pH modulation, and secreted fungal metabolites such as farnesol that alter *S. aureus* stress responses and antimicrobial tolerance. Together, these interactions illustrate how metabolic and environmental cross-talk can enhance *S. aureus* pathogenicity during co-infections and highlight potential targets for therapeutic intervention in polymicrobial infections.

## QS-related and host-mediated interactions during *S. aureus-C. albicans* coinfection

Host innate immunity represents an additional interface through which *C. albicans-S. aureus* interactions can influence infection outcomes. Highly immunogenic *C. albicans* hyphae attract phagocytic cells that preferentially engulfed hyphae-associated *S. aureus*. In a murine oral coinfection model, phagocyte-associated *S. aureus* was subsequently trafficked to draining cervical lymph nodes and distal organs, supporting a host-mediated mechanism of bacterial dissemination [58]. Separately, farnesol has been shown to stimulate macrophage chemokinesis in vitro and to increase macrophage accumulation in the murine peritoneal cavity following intraperitoneal administration [59], although a direct role for farnesol in the bait-and-switch dissemination mechanism has not been demonstrated.

Farnesol can also modulate macrophage inflammatory responses in conjunction with fungal cell-wall stimuli. In RAW264.7 macrophages, farnesol synergised with zymosan, a yeast cell-wall preparation containing fungal pathogen-associated molecular patterns (PAMPs), to enhance IL-6 expression [60]. The combination of zymosan and farnesol also increased Dectin-1 expression, whereas TLR2 expression was induced by zymosan alone and was not further enhanced by farnesol [60]. In primary human neutrophils, farnesol promotes oxidative burst and other features of low-grade activation [61]. At higher concentrations used in vitro (100-200 μM), farnesol also promotes NET formation through a ROS-dependent pathway involving Mac-1 (CD11b/CD18), TLR2, PKC, ERK1/2, and NADPH oxidase [62].

Farnesol additionally exerts immunomodulatory effects on adaptive immune responses. In a murine model of systemic candidiasis, exogenous farnesol pretreatment reduced serum IFN-*γ* and IL-12 while increasing IL-5, suggesting suppression of protective Th1-type responses with a possible Th2-skewing effect [63]. Consistently, in human monocyte-derived dendritic cells, farnesol impaired dendritic-cell differentiation, reduced IL-12 production, and compromised the ability of dendritic cells to stimulate T-cell responses [61]. Together, these findings indicate that farnesol exerts context-dependent and pleiotropic effects on host immunity rather than uniformly activating or suppressing immune responses.

Concurrently, the *S. aureus* agr QS interacts with the host innate immunity through both agr-regulated virulence factors and reciprocal host-mediated inhibition of agr signalling. AgrA directly activates transcription of the psmα operon independently of RNAIII [33]. Agr-regulated PSMα peptides contribute to *S. aureus* survival within neutrophils, whereas Agr activity limits late-endosomal localisation and promotes phagosomal escape into the cytosol [64]. PSMα peptides can also induce a rapid, non-classical form of NET formation that is independent of FPR2 receptor, myeloperoxidase (MPO), neutrophil elastase, and NADPH oxidase-derived reactive oxygen species (ROS) [65]. This process can occur at 4°C, suggesting that membrane-disturbing activity of PSMα may contribute to NET release rather than conventional receptor-mediated signalling [65]. Although PSMα-induced NETs can capture microbes [65], excessive NET formation can also contribute to tissue damage in inflammatory settings [66]; thus, the net contribution of PSMα-induced NET formation to *S. aureus* pathogenesis in vivo remains unresolved (Figure 1).

AgrD processing also generates bioactive N-terminal AgrD (N-AgrD) peptides in addition to mature AIP. The formylated N-AgrD F20 and F24 variants exhibit potent FPR-dependent neutrophil chemoattractant activity, whereas N-AgrD F20 specifically induces NET formation [67].

Host immunity can, in turn, directly interfere with agr signalling. Phagocyte-derived oxidants, including HOCl and ONOO^−^, oxidise the C-terminal methionine of type I AIP and abolish its signalling activity [68]. Oxidative stress can also regulate agr intracellularly by inducing a Cys199-Cys228 disulphide switch in AgrA, thereby reducing its DNA-binding activity and altering agr-dependent transcription [69]. Additional host-derived factors suppress agr through distinct mechanisms: serum apolipoprotein B sequesters AIP and prevents agr activation, whereas haemoglobin reduces agr P3 promoter activity [5].

Together, these findings illustrate a bidirectional interface between microbial QS pathways and host immunity. Microbial QS-related factors, including fungal farnesol and agr-regulated bacterial peptides, can alter phagocyte behaviour through distinct mechanisms, whereas host-derived oxidants and serum factors can directly restrict bacterial QS signaling [5,59,60,62,65,67–69]. These reciprocal interactions highlight host–QS interfaces as potential therapeutic targets, although their context-dependent effects on antimicrobial defence and immune homoeostasis require careful evaluation before therapeutic translation (Table 1).

**Table 1.** Principal mechanistic categories of *Candida albicans-Staphylococcus aureus* cross-kingdom interactions and supporting experimental evidence.

Principal interspecies mechanism	Signalling molecule(s)/pathway(s) involved	Experimental model	Biological effect	Supporting reference(s)
**Metabolic regulation of bacterial QS**	*C. albicans* Zcf13p-RBK1/HGT7-dependent ribose utilisation → extracellular ribose depletion → relief of ribose-mediated repression of the *S. aureus* agr system	*C. albicans-S. aureus* coculture using WT, *zcf13Δ/Δ*, *rbk1Δ/Δ*, and complemented strains; murine polymicrobial intra-abdominal infection	Ribose depletion enhances agr activation and *α*-toxin production; restoration of RBK1/HGT7-dependent ribose utilisation restores polymicrobial pathogenicity	[1]
**Environmental modulation of bacterial QS**	Stp2-dependent amino-acid utilisation/ammonia-mediated extracellular pH modification → pH-sensitive agr regulation	In vitro *C. albicans-S. aureus* coculture with alkalinization-deficient *C. albicans* mutants and pH manipulation; comparison among *Candida* species; murine IAI used to assess in vivo relevance	Fungal alkalinization toward a permissive pH can sustain agr activity and *α*-toxin production in vitro; the effect is context dependent and alkalinization alone is insufficient to explain lethal synergy in vivo	[1,50,53,70]
**Structural biofilm interaction**	*C. albicans* hyphal scaffold; hypha-associated adhesion; extracellular matrix/eDNA	In vitro dual-species biofilms; microscopy, adhesion assays, ECM-degradation experiments	Preferential attachment of *S. aureus* to *C. albicans* hyphae promotes spatial organisation and bacterial colonisation within mixed biofilms; fungal matrix components further support biofilm persistence	[41,71]
**Farnesol-mediated cross-kingdom effects**	*C. albicans*-derived farnesol → bacterial oxidative-stress/redox responses and efflux-associated adaptation; fungal morphogenesis inhibition	In vitro *S. aureus* biofilms and sequential farnesol exposure; mixed *C. albicans–S. aureus* cultures/biofilms; comparison with farnesol-deficient *C. albicans*	Concentration- and exposure-dependent remodelling of mixed biofilms; increased antimicrobial tolerance/efflux in *S. aureus*; reduced staphyloxanthin and altered oxidative-stress adaptation	[42,46,47]
**Fungal metabolite-mediated stimulation of bacterial biofilm growth**	*C. albicans*-derived prostaglandin E₂ (PGE₂)	In vitro mixed biofilms; purified PGE₂; cell-free fungal supernatants; PGE₂-deficient *C. albicans* mutant; cyclooxygenase inhibition	PGE₂ promotes *S. aureus* growth and biofilm formation in the mixed-biofilm setting	[48]
**Host-mediated polymicrobial dissemination**	*C. albicans* hyphae/hypha-associated *S. aureus* → phagocyte recruitment and uptake	Time-lapse macrophage–biofilm assays; immunosuppressed murine oral coinfection model	Phagocytes preferentially interact with *C. albicans* hyphae, engulf adherent *S. aureus*, and can facilitate bacterial dissemination from the mucosal site	[58]
**QS-related modulation of host innate immunity**	Farnesol; Agr-regulated PSMα peptides; N-terminal AgrD peptides	Murine macrophage assays and selected animal models; isolated human neutrophils; NET-formation and chemotaxis assays	Modulation of macrophage recruitment/activation, neutrophil responses and NET formation; the direction and magnitude of these effects depend on the microbial factor and experimental context	[59–65,67]
**Host feedback on bacterial QS**	Host-derived oxidants (HOCl/ONOO^-^, oxidative stress) → AIP oxidation/AgrA redox regulation; ApoB-mediated AIP sequestration; haemoglobin-mediated agr repression	Biochemical and in vitro agr assays; neutrophil/host-factor studies	Host-derived factors can suppress agr signalling at the level of AIP, AgrA, or agr transcription, providing reciprocal host control of bacterial QS activity	[5,68,69]

Direct heterologous recognition of a *C. albicans*-derived quorum-sensing molecule by a defined *S. aureus* QS receptor has not yet been demonstrated. Accordingly, metabolic, environmental, structural, secreted-metabolite, and host-mediated interactions are distinguished here from direct interkingdom QS signalling.

**Figure 1.**
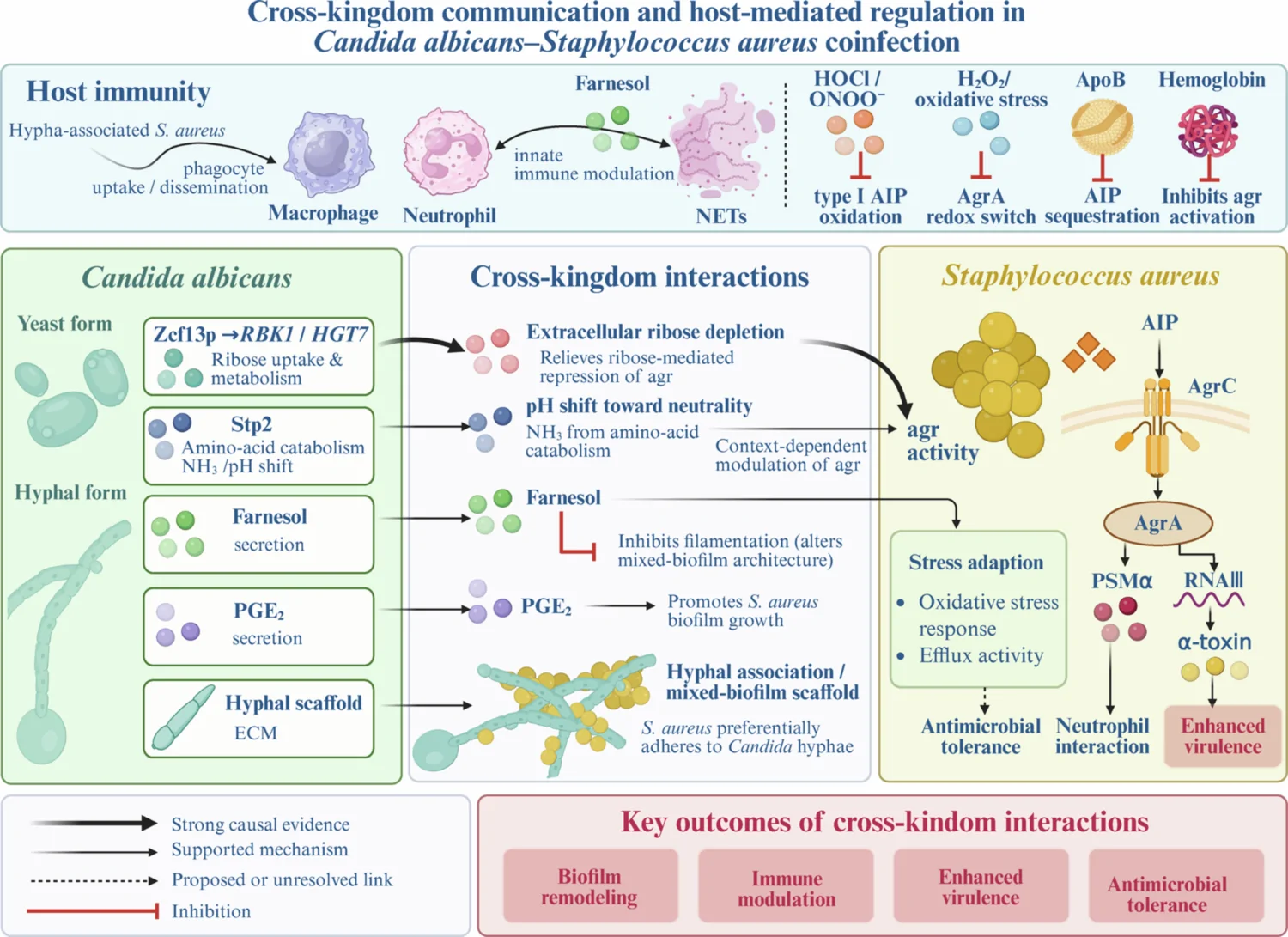
Cross-kingdom communication and host-mediated regulation during *Candida albicans-Staphylococcus aureus* coinfection. Multiple metabolic, environmental, structural, secreted-metabolite, and host-mediated mechanisms contribute to *C. albicans-S. aureus* interactions. In *C. albicans*, the transcription factor Zcf13p regulates the downstream genes *RBK1* and *HGT7*, promoting ribose uptake and metabolism. Depletion of extracellular ribose relieves ribose-mediated repression of the *S. aureus* agr system, resulting in enhanced agr activity and downstream virulence; this pathway is supported by strong causal evidence from coculture and murine coinfection models. Stp2-dependent amino-acid catabolism and NH_3_ production can shift extracellular pH toward neutrality and modulate agr activity in a context-dependent manner. Farnesol inhibits *C. albicans* filamentation and thereby remodels mixed-biofilm architecture, while also altering *S. aureus* stress-adaptation responses, including oxidative-stress responses and efflux activity, with reduced staphyloxanthin production observed as a parallel phenotype. PGE_2_ promotes *S. aureus* biofilm growth, whereas *C. albicans* hyphae and extracellular matrix provide a structural scaffold that facilitates bacterial association within mixed-species biofilms. Within the *S. aureus* agr circuit, AIP-mediated activation of AgrC and AgrA regulates RNAIII-dependent *α*-toxin production and directly controls PSMα expression, linking agr activity to enhanced virulence and neutrophil interactions. At the host interface, phagocytic uptake of hypha-associated *S. aureus* can facilitate bacterial dissemination, while farnesol modulates innate immune responses and NET formation. Conversely, host-derived factors can suppress agr signalling through oxidation of type I AIP by HOCl/ONOO^-^, oxidative-stress-induced redox regulation of AgrA, sequestration of AIP by apolipoprotein B (ApoB), and inhibition of agr activation by haemoglobin. Collectively, these interactions contribute to biofilm remodelling, immune modulation, enhanced virulence, and antimicrobial tolerance. Thick solid arrows indicate mechanisms supported by strong causal evidence; Thin solid arrows indicate supported mechanisms; dashed arrows indicate proposed or incompletely resolved links; red blunt-ended lines indicate inhibition. Direct heterologous recognition of a *C. albicans*-derived quorum-sensing molecule by a defined *S. aureus* QS receptor has not yet been demonstrated.

## Therapeutic strategies targeting Qs-related and cross-kingdom interactions

Therapeutic strategies targeting the *S. aureus* QS system are characterised by multi-level, targeted interventions. The core paradigm centres on Quorum Quenching (QQ) to obstruct virulence signal transduction without exerting direct bactericidal pressure. This antivirulence strategy may impose less selective pressure than conventional bactericidal approaches [72–74]. Precise intervention involves the comprehensive disruption of signalling molecule synthesis, receptor recognition, and downstream effector pathways. At the level of AIP maturation, AgrB-mediated C-terminal cleavage and cyclization of AgrD represent potential intervention points for disrupting AIP production [72]. At the membrane interface, AgrC-mediated signalling can be antagonised by AIP-like molecules and natural products. Solonamide B acts as an antagonist of AgrC-mediated signalling, whereas *Hydrastis canadensis* leaf extracts attenuate AgrCA-dependent signalling, although their precise molecular target remains unresolved [75,76]. At the transcriptional regulation level, small molecules such as savirin and *ω*-hydroxyemodin inhibit AgrA-dependent transcription by interfering with AgrA binding to promoter DNA, thereby suppressing P2/P3-driven agr activity and downstream virulence gene expression [73,77]. Carnosic acid and carnosol, two major constituents of rosemary leaves, inhibit AIP-induced RNAIII transcription and psmα expression [74]. Staquorsin similarly suppresses RNAIII and agr-regulated virulence phenotypes and has shown efficacy in a murine skin infection model, although its direct interaction with AgrA is supported primarily by in silico docking [78]. The RIP/RAP-TRAP axis has also been explored therapeutically: RIP was reported to inhibit RAP-induced TRAP phosphorylation, while RIP-V reduced RNAIII expression and *α*-hemolysin release and improved survival in a murine MRSA sepsis model [79,80].

In the context of fungal QS regulation, farnesol has emerged as a pleiotropic molecule with therapeutic potential. Farnesol suppresses the yeast-to-hypha transition of *C. albicans* and affects Ras1-Cyr1-cAMP/PKA-dependent signalling [23,39]. In biofilm models, farnesol also reduces *CYR1* expression and intracellular cAMP levels while modulating *PDE2* expression, changes associated with increased antifungal susceptibility [81]. In parallel, farnesol inhibits hyphal initiation by interfering with Ubr1-dependent Cup9 degradation, thereby repressing *SOK1* expression and preventing Nrg1 protein degradation [82]. Notably, farnesol generally dominates over tyrosol in regulating *C. albicans* morphogenesis, although high concentrations of tyrosol can counteract the biofilm-inhibitory effect of relatively low concentrations of farnesol [83]. Farnesol can also synergise with fluconazole and other antifungal agents by modulating ABC transporter-mediated drug efflux and promoting ROS accumulation and early apoptosis, including in azole-resistant *C. albicans* isolates [84].

Therapeutic strategies targeting quorum sensing (QS) are increasingly being combined with complementary antimicrobial approaches (Table 2). Probiotic-derived signalling molecules provide one example: cyclic dipeptides produced by *Lactobacillus reuteri* RC-14 suppress *S. aureus* agr P2/P3 transcription and reduce agr-regulated toxin production [85]. Nanomaterial-based antivirulence approaches have also been explored. Silver-metformin nanoformulations reduce several *S. aureus* virulence-associated phenotypes and the expression of genes including *crtM, sigB, sarA,* and *fnbA*; however, *agrA* itself was upregulated under the tested conditions, indicating that these effects should not be interpreted as simple suppression of the agr pathway [86]. In parallel, multifunctional delivery platforms can integrate QS interference with direct antimicrobial activity. The BBR@SP hydrogel combines berberine-mediated QS interference with laser-triggered ROS production and biofilm disruption and showed therapeutic efficacy in a murine MRSA-infected diabetic wound model [87]. Collectively, these preclinical approaches illustrate the potential value of combining antivirulence strategies with complementary antimicrobial mechanisms rather than relying on QS inhibition alone.

In polymicrobial infections, particularly those involving *S. aureus* and *C. albicans*, cross-kingdom therapeutic strategies that affect both organisms are beginning to emerge. Farnesol exemplifies this concept by suppressing *C. albicans* filamentation while increasing the susceptibility of *S. aureus* to selected antibiotics. In mixed biofilms, farnesol combined with oxacillin achieved substantial biofilm inhibition, and a pilot FISH analysis showed loss of detectable staphylococcal activity after combined treatment [42]. Beyond endogenous fungal metabolites, plant-derived shikonin has shown dual-species antibiofilm and antivirulence activity: it downregulated *agrA* and RNAIII in *S. aureus* while inhibiting hyphal development in *C. albicans*, suppressed polymicrobial biofilms development in vitro and ex vivo, and reduced microbial burden in a murine *S. aureus-C. albicans* wound infection model [88]. Coumarins and their derivatives have also demonstrated QS-inhibitory and antibiofilm activities against multiple microbial species, including *S. aureus* and *C. albicans*; however, current evidence does not establish simultaneous inhibition of defined agr and fungal QS pathways within *S. aureus-C. albicans* mixed biofilms [89]. Together, these findings support the feasibility of coordinated cross-kingdom intervention, while direct simultaneous targeting of defined QS pathways in both organisms remains to be demonstrated.

**Table 2.** Representative experimental therapeutic strategies targeting QS-related pathways in *Staphylococcus aureus* and *Candida albicans* and cross-kingdom pathogenic interactions.

Category	Target/intervention	Mechanistic basis	Experimental model/evidence level	Reported biological effect	Reference(s)
***S. aureus*** **agr-targeted strategies**					
AIP maturation	AgrB/AgrD processing	Interference with AIP precursor processing represents a potential strategy to reduce mature AIP production	Mechanistic/in vitro	Potential inhibition of agr activation	[72,90]
Receptor recognition	AgrC; Solonamide B	Antagonises AIP-mediated AgrC activation	in vitro; ex vivo cell-based assays	Reduced agr signalling and virulence-associated phenotypes	[76]
Transcriptional regulation	AgrA; *ω*-hydroxyemodin, savirin	Interferes with AgrA-dependent DNA binding and transcriptional activation	In vitro+animal models	Reduced RNAIII/PSM/virulence-factor expression	[73,77]
agr-regulated transcription	Carnosic acid/carnosol	Inhibits AIP-induced RNAIII and psmα expression	In vitro	Attenuated agr-regulated virulence	[74]
Historical agr-interference strategy	RIP	Proposed interference with RAP/TRAP-associated regulation	In vitro+animal studies; historical model	Reduced RNAIII-associated virulence phenotypes	[79,80]
***C. albicans*** **QS-related strategies**					
Morphogenetic signalling	Farnesol	Modulates Ras1-Cdc35-PKA signalling and inhibits hyphal morphogenesis	In vitro	Reduced yeast-to-hypha transition	[39]
Hyphal initiation	Farnesol/Ubr1-Cup9-Sok1-Nrg1 axis	Prevents Nrg1 degradation through Ubr1-dependent regulation	In vitro	Inhibition of hyphal initiation	[82]
Morphogenesis modulation	Farnesol+tyrosol	Concentration-dependent antagonistic interaction between fungal signalling molecules	In vitro	Modulation of hyphal/biofilm development	[83]
Antifungal combination	Farnesol+fluconazole	Farnesol modulates drug efflux and can enhance antifungal activity	In vitro	Increased antifungal susceptibility/enhanced drug activity	[84]
Biofilm-associated signalling	Farnesol; CYR1/PDE2-associated regulation	Modulates cAMP-associated signalling in biofilms	In vitro	Reduced biofilm-associated antifungal tolerance	[81]
**Multimodal and dual-species strategies**					
Interspecies agr inhibition	*Lactobacillus reuteri* RC-14 cyclic dipeptides	Suppresses agr P2/P3 transcription and agr-regulated toxin expression	In vitro	Reduced agr-dependent virulence-factor production	[85]
Multimodal antimicrobial platform	BBR@SP hydrogel	Combines QS-related interference with laser-triggered ROS generation	In vitro+murine infected-wound model	Reduced biofilm/virulence and improved wound healing	[87]
Dual-species combination	Farnesol+oxacillin	Combines fungal morphogenesis inhibition with increased staphylococcal *β*-lactam susceptibility	In vitro mixed-biofilm model	Substantial inhibition of mixed biofilms	[42]
Dual-species antivirulence strategy	Shikonin	Downregulates agr-associated genes in *S. aureus* and inhibits *C. albicans* hyphal development	In vitro multispecies biofilm+murine coinfection model	Reduced multispecies biofilm formation and microbial burden	[88]

## Conclusions

Although substantial mechanistic evidence supports cross-kingdom interactions between *C. albicans* and *S. aureus*, the available studies are highly heterogeneous with respect to experimental model, microbial strain, nutrient composition, farnesol concentration, and infection site. These variables can substantially influence fungal morphogenesis, bacterial agr activity, biofilm architecture, antimicrobial tolerance, and host responses, and may partly account for apparently divergent findings across studies. Consequently, mechanisms established in a particular in vitro or animal model should not be assumed to operate with the same magnitude or direction in other host environments, particularly the human oral cavity.

In summary, accumulating evidence indicates that the synergistic pathogenicity associated with *S. aureus* and *C. albicans* co-infection is closely linked to multiple cross-kingdom interactions that include, but are not limited to, QS-related regulation. The strongest mechanistic evidence supports metabolic regulation of the *S. aureus* agr system through *C. albicans*-mediated ribose depletion, structural interactions within mixed biofilms, and farnesol-mediated modulation of bacterial stress responses and antimicrobial tolerance. Environmental and host-mediated effects also contribute to these interactions, although their magnitude and relevance are highly context dependent. Importantly, direct heterologous recognition of a *C. albicans*-derived QS molecule by a defined *S. aureus* QS receptor has not yet been demonstrated.

Targeting these interactions therefore represents a promising therapeutic direction for refractory polymicrobial infections. Strategies such as quorum-quenching approaches, autoinducing peptide analogues, farnesol-based interventions, and anti-adhesion approaches may provide opportunities to disrupt pathogenic interactions with less direct bactericidal pressure than conventional antimicrobial agents. However, these approaches remain predominantly experimental and preclinical, and their efficacy and potential for resistance selection require evaluation. Validation in physiologically relevant oral and systemic infection models remains necessary before clinical translation.

Future research should prioritise direct validation of interspecies signal recognition and the dynamic characterisation of cross-species interactions. A particular gap is the lack of host-relevant oral models that adequately reproduce the spatial organisation, nutritional conditions, and immune complexity of the oral cavity. Particular attention should also be given to strain-, nutrient-, concentration-, and infection-site-dependent heterogeneity, as these factors may substantially alter the direction and magnitude of interspecies effects. A deeper understanding of these interactions may ultimately contribute to more effective management strategies for complex polymicrobial diseases.

## Data Availability

Data sharing is not applicable to this article as no new data were created or analysed in this study.
